# Simultaneous Chronoamperometric Sensing of Ascorbic Acid and Acetaminophen at a Boron-Doped Diamond Electrode

**DOI:** 10.3390/s8063952

**Published:** 2008-06-12

**Authors:** Codruţa Cofan, Ciprian Radovan

**Affiliations:** 1 University of Medicine and Pharmacy “Victor Babeş” Timişoara, Piaţa E. Murgu, Nr. 2, 300041 Timişoara, Romania; 2 West University of Timişoara, Laboratory of Electrochemistry, Str. Pestalozzi Nr.16, 300115 Timişoara, Romania; E-mail: radovan@cbg.uvt.ro (C.R.)

**Keywords:** acetaminophen, ascorbic acid, chronoamperometry, boron-doped diamond electrode, buffered media

## Abstract

Cyclic voltammetry (CV) and chronoamperometry (CA) have been used to sense and determine simultaneously L-ascorbic acid (AA) and acetaminophen (AC) at a boron-doped diamond electrode (BDDE) in a Britton-Robinson buffer solution. The calibration plots of anodic current peak versus concentration obtained from CV and CA data for both investigated compounds in single and di-component solutions over the concentration range 0.01 mM – 0.1 mM proved to be linear, with very good correlation parameters. Sensitivity values and RSD of 2-3% were obtained for various situations, involving both individual and simultaneous presence of AA and AC. The chronoamperometric technique associated with standard addition in sequential one step and/or two successive and continuous chronoamperograms at two characteristic potential levels represented a feasible option for the simultaneous determination of AA and AC in real sample systems such as pharmaceutical formulations. The average values indicated by the supplier were confirmed to a very close approximation from chronoamperomgrams by using several additions with the application of suitable current correction factors.

## 1. Introduction

L-Ascorbic acid (L-threo-hex-2-enono-1, 4-lactone or Vitamin C), hereafter referred to as AA, a water soluble vitamin, is an extremely important substance which plays a unique redox and electrochemical role [1]. AA is a compound that takes part in many important life processes [2, 3]. Due to its antioxidant and pH regulator properties, this vitamin is present or added to a wide variety of food products and pharmaceuticals. Ascorbic acid is easily oxidized chemically and electrochemically to L-dehydroascorbic acid [4]. AA is unstable, undergoing oxidation, especially in aerobic conditions, alkaline media, and at exposure to light [5]. Ascorbic acid is widely found in association with various biologically and pharmacologically active substances, including acetaminophen [6] in various pharmaceutical products as well as in biological fluids [6-9]. Attributed to the complementary pharmacological character of the associated substances, there has been a growing interest in the investigation of such multi-component systems.

Several available methods have been reported for the determination of ascorbic acid in pharmaceutical preparations, biological fluids, food and beverages. The methods are: titration, fluorimetry, high performance liquid chromatography, spectrophotometry, UV and even electrochemical detection in single component or multi-component systems using a whole range of electrodes [6, 10-16].

Acetaminophen (AC) (N-acetyl-*p*-aminophenol) also known as paracetamol is an extensively employed antipyretic analgesic frequently prescribed on its own or combined with other related drugs [17]. AC is the component of a number of analgesic pharmaceutical preparations, both single drug one and multidrug formulations. Although it is known that paracetamol has no anti-inflammatory properties, its action is similar to acetylsalicylic acid [18]. AC represents an attractively alternative for patients who are sensitive to aspirin and it is the analgesic of choice for people with asthma [19]. Acetaminophen is metabolized predominantly in the liver and excreted in the urine mainly as the glucuronide and sulphate conjugates [20]. However, many studies have shown that overdose of acetaminophen is associated with hepatic toxicity and renal failure despite its apparently innocuous character [21].

In therapeutic use, acetaminophen is often found associated with ascorbic acid and/or other pharmacologically and biologically active compounds [22, 23]. The adjunct presence of ascorbic acid to acetaminophen leads to diminished toxicity and intensified positive effects of acetaminophen [24, 25].

To date, a large number of methods have been proposed for the analysis of acetaminophen in pharmaceutical formulations, biological fluids, and even wastewaters [26]. Titrimetric analysis, spectrofluorimetry, spectrophotometry, various kinds of high performance liquid chromatography, capillary electrophoresis conducted in single component as well as multi-component systems have to be mentioned [21, 27-29]. Due to its electroactive properties, acetaminophen has been also investigated by various electrochemical methods performed in a variety of supporting electrolytes and using different kinds of electrodes [21, 30-40], including the boron-doped diamond electrode (BDDE) [41].

The excellent combination of mechanical, thermal, and chemical properties of diamond makes this material an ideal choice for a large number of applications in aggressive media. Boron doped diamond (BDD) electrodes have received much attention due to the very wide electrochemical window in aqueous solutions resulting from the high overpotentials of the oxygen and hydrogen evolution reactions and the low reactivity of their surface. BDD electrodes also exhibit a low and stable voltammetric and amperometric background current and good activity towards some redox analytes without any conventional pretreatment [42-49].

Acetaminophen as well as ascorbic acid has already been electrochemically detected in the presence of other active substances using different types of electrodes and several supporting electrolytes [37, 50]. The compounds mentioned have also been investigated individually in single component systems at a BDDE by cyclic voltammetry and flow injection analysis with amperometric detection in supporting electrolytes as phosphate buffer or NaClO_4_ in an ethanol-water solution [41, 48].

Electrochemical oxidation of acetaminophen, including a detailed description of the reaction mechanism variants, has been reported by Kissinger and co-workers [51, 52]. Several reports have presented electroanalytical data, involving the determination of AA and AC present simultaneously in aqueous mixture solutions [6, 30, 31], using carbon paste electrodes and glassy carbon electrode. To our knowledge, no other work has reported the application of a commercial BDDE for the simultaneous detection of AA and AC, except for our previous paper [53].

This paper presents a new, detailed and explicit investigation of the concomitant assessment of ascorbic acid and acetaminophen by cyclic voltammetry and proposes an analytical application in original manner of the simultaneous anodic chronoamperometric sensing for the determination of AA and AC at a stationary unmodified boron-doped diamond electrode using as supporting electrolyte an acidic Britton-Robinson buffer solution, a selected medium which assured a sharp current peaks separation. Finally, the actual report, presented for the simultaneous determination of AA and AC contents in two pharmaceutical preparations, furnishes evidence of the simplicity and the practical utility of the proposed chronoamperometric sensing and the assessment method associated with standard addition. The chronoamperometric alternative itself could be adapted for various sensing circumstances, *e.g.* in metabolic fluids, or in flowing systems.

## 2. Experimental Section

All electrochemical measurements were performed in a Metrohm-three electrode cell. The working electrode employed as amperometric sensor was an unmodified commercial boron-doped diamond electrode supplied by Windsor Scientific Ltd. for electroanalytical use. The counter-electrode was a platinum foil, and the reference was a saturated calomel electrode (SCE). The mirror-polished doped polycrystalline industrial diamond (microcrystalline doping degree ∼ 0.1% boron) was provided as a 3 mm-diameter stationary disc embedded in a Teflon rod [49, 53], previously stabilized in our laboratory by mild electrochemical oxidation and several hundreds repeated alternate polarizations by cycling between + 1.8V and -1V vs. SCE potential limits in neutral sodium sulphate supporting electrolyte. An Autolab PGStat 20 EcoChemie system controlled by a PC running GPES Software version 4.8 was used for collecting the cyclic voltammograms (CVs) and chronoamperograms (CAs). Working parameters for the exemplified cyclic voltammograms involved a scan rate of 0.05 Vs^-1^ and a potential range between 0V and + 1.25 V vs. SCE. CVs and CAs were recorded at stationary electrode, in quiescent solutions, in a controlled argon atmosphere, and at room temperature (23±1°C). The final solution volume in the cell was 50 mL.

Before starting each series of electrochemical measurements, the working electrode was cleaned, degreased**,** simply polished on a wetted pad mostly without powder, and only rarely with alumina aqueous suspension after other utilizations and carefully washed with double distilled water. Each determination was repeated three times with good reproducibility of the practically stabilized state of electrode surface recovered by simple means of a short rest period between measurements combined with brief stirring of the solution without any supplementary cleaning of the electrode between the successive measurements.

A relatively concentrated Britton-Robinson buffer, pH 1.96, with no supplementary salt addition, containing 0.04 M of each component in the mixture solution was used as supporting electrolyte. The investigated substances were analytical grade Fluka and Merck reagents. Ascorbic acid and acetaminophen standard solutions were freshly prepared and the explored concentrations of these compounds for the delimitation of a coherent electroanalytical method ranged between 0.01-0.1 mM in cyclic voltammetry experiments and 0.01-0.07 mM in chronoamperometric exploration. The higher investigated concentration ranges had no relevance for electroanalytical purpose, but a distinct access to a low limit of detection was desirable. The suitability of the chronoamperometric method for the simultaneous detection and determination of the investigated components was verified and certified by chronoamperograms (CAs) recorded in association with standard addition method using pharmaceutical formulations, containing AA and AC as real samples. The aqueous real sample solutions were obtained from two UPSA products, Efferalgan effervescent tablets and Fervex powder doses. 500mL as stock aqueous solutions were made up from a single Efferalgan tablet or one Fervex dose. Daily-prepared AA and AC standard solutions and Efferalgan and Fervex solutions were used. The suitability of the chronoamperometric method for obtaining a fast and direct overall amperometric characteristic of a solution series from the same mixture product has been only implicitly tested.

## 3. Results and Discussion

### 3.1. Cyclic anodic voltammetry data

**Figure 1.**
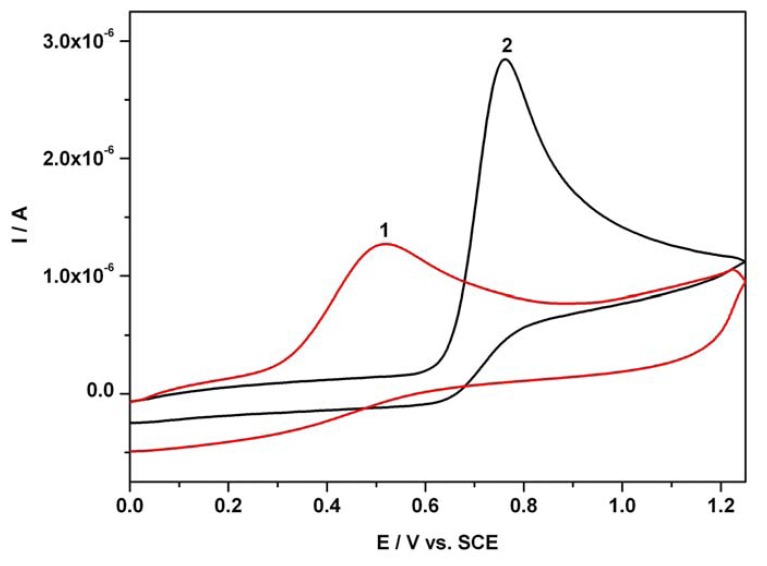
Cyclic voltammograms (CVs); 1 - 0.06 mM AA; 2 - 0.12 mM AC; supporting electrolyte: Britton-Robinson buffer pH 1.96; starting potential 0V vs. SCE; potential range: 0 V → + 1.25 V → 0 V vs. SCE; scan rate: 0.05 Vs^-1^.

In order to establish the potential steps used in our chronoamperometric study concerning the determination of AA and AC in individual and mixture systems, as well as the linearity of the amperometric signal versus concentration, the distinct and resultant cyclic voltammograms (CVs) for both investigated compounds were recorded. Figure 1 shows two examples of distinct anodic cyclic voltammetric responses (first scan) corresponding to electrochemical oxidation of 0.06 mM AA and 0.12 mM AC in single component solution at an unmodified BDDE in an acidic Britton-Robinson buffer solution. The CVs were recorded in a potential range between 0V and +1.25 V vs. SCE starting in the positive direction from 0V vs. SCE at a scan rate of 0.05 Vs^-1^. The anodic current peaks attributed to AA and AC were manifested on the forward branches of CVs at potentials around + 0.5 V vs. SCE, and + 0.75 V vs. SCE, respectively.

**Figure 2.**
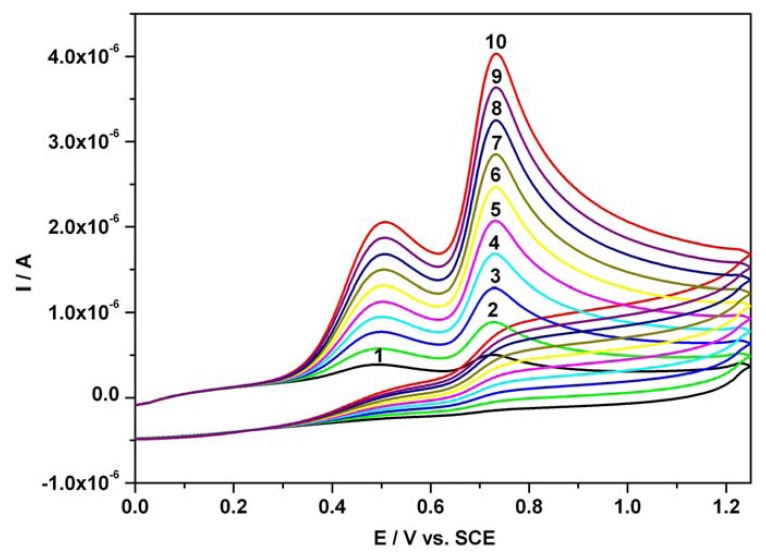
Resultant cyclic voltammograms (CVs) of AA and AC mixture; 1 – 0.01 mM AA and 0.01 mM AC; 2 – 0.02 mM AA and 0.02 mM AC; 3 – 0.03 mM AA and 0.03 mM AC; 4 – 0.04 mM AA and 0.04 mM AC; 5 – 0.05 mM AA and 0.05 mM AC; 6 – 0.06 mM AA and 0.06 mM AC; 7 – 0.07 mM AA and 0.07 mM AC; 8 – 0.08 mM AA and 0.08 mM AC; 9 – 0.09 mM AA and 0.09 mM AC; 10 – 0.1 mM AA and 0.1 mM AC; supporting electrolyte: Britton-Robinson buffer pH 1.96; starting potential 0V vs. SCE; potential range: 0 V → + 1.25 V → 0 V vs. SCE; scan rate: 0.05 Vs^-1^.

A series of resultant CVs obtained directly over the concentration range 0.01 mM - 0.1 mM for the mixture of ascorbic acid and acetaminophen standard solutions, corresponding to an additive behaviour of CVs, is illustrated in Figure 2. At equal concentrations of both compounds in Britton-Robinson buffer medium, two well-defined, separated current peaks manifested for AA and AC practically at the same specific potential values as in the single substance studies. This observation led to the assumption that chronoamperometry at two distinct potential levels could represent another and complementary alternative related to our previous investigation for direct sensing and the simultaneous determination of AA and AC at a BDDE in a simply prepared acidic Britton-Robinson buffer used as supporting electrolyte.

**Figure 3.**
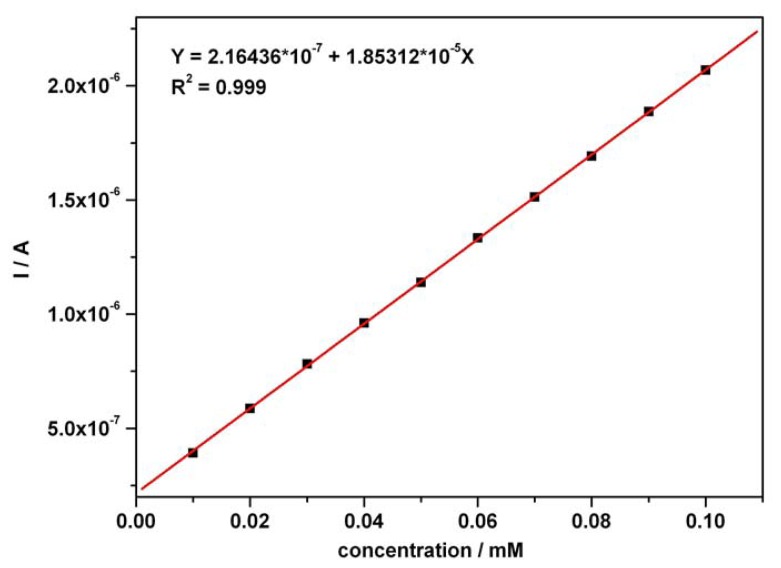
Calibration plot (with supporting electrolyte – background current correction) of I vs. AA concentration in mixture solution at current peak potential around + 0.5 V vs. SCE (under the conditions mentioned in Figure 2).

**Figure 4.**
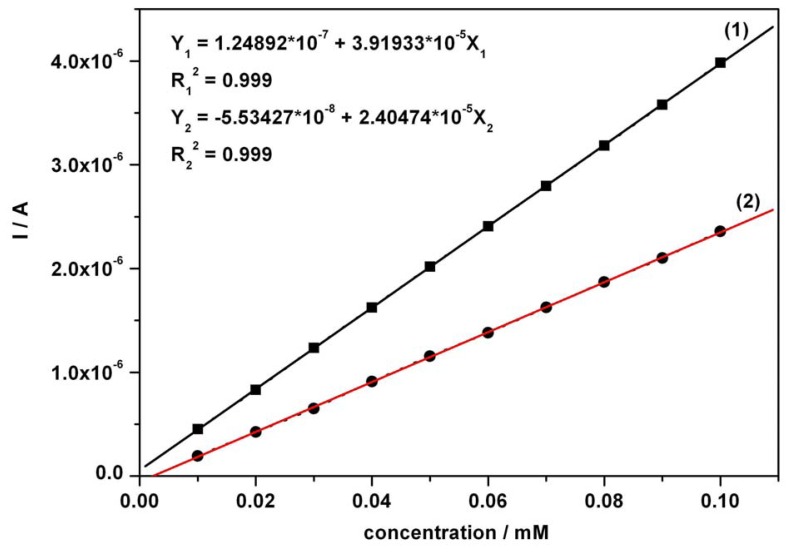
Calibration plots of I vs. AC concentration in mixture solution around + 0.75 V vs. SCE (under the conditions mentioned in Figure 2); (1) – without background current correction; (2) – approximated with a subtraction of extrapolated AA current read around + 0.625 V vs. SCE, as a correction for AC current peak values.

The calibration plots (Figures 3 and 4) of anodic current peaks versus concentration of AA as well as AC in the mixture system, were linear exhibiting very good correlation coefficients and high sensitivities of 18.53 μA mM^-1^ for AA (curve in Figure 3) and 24.05 μA mM^-1^ for AC (curve 2 in Figure 4); curve 2 has been derived from curve 1 by an approximated current correction through AA remnant current in the potential range corresponding to AC detection. The obtained sensitivity from curve 2 was below 39.19 μA mM^-1^, the apparent sensitivity without any correction (curve 1 in Figure 4). A corrected sensitivity for AC has been evaluated by approximated extrapolation of remnant current derived from the minimum values read on the forward branches of the CVs from Figure 2 around 0.625-0.65 V vs. SCE. The essential aspects regarding direct linearity of the resultant amperometric signals versus concentration were determined and certified to be in accordance with our previous conclusions [53]. The obtained CV data and the very good linearity of the plots of current peaks versus concentration for the used compounds suggested a very useful foundation for the next step as practical applications of the chronoamperometric sensing and evaluation of the target compounds concentrations at established optimum fixed potential.

### 3.2. Chronoamperometry data

After the preliminary discussion of cyclic voltammetry data, a more detailed investigation was conducted using the chronoamperometry (CA) method to establish several working conditions and calibration plots data available as support for application in practical purposes. The chronoamperometric results were collected for both AA and AC in individual and mixture solution. The continuous chronoamperograms reported here were obtained in quiescent solution. This combined method has been elaborated in original manner for a di-component system and regarded as a modified and adapted variant of the use of multipotential levels continuous CAs resulted in the amperometric analytical evaluation of a multistep oxidation system [49].

**Figure 5.**
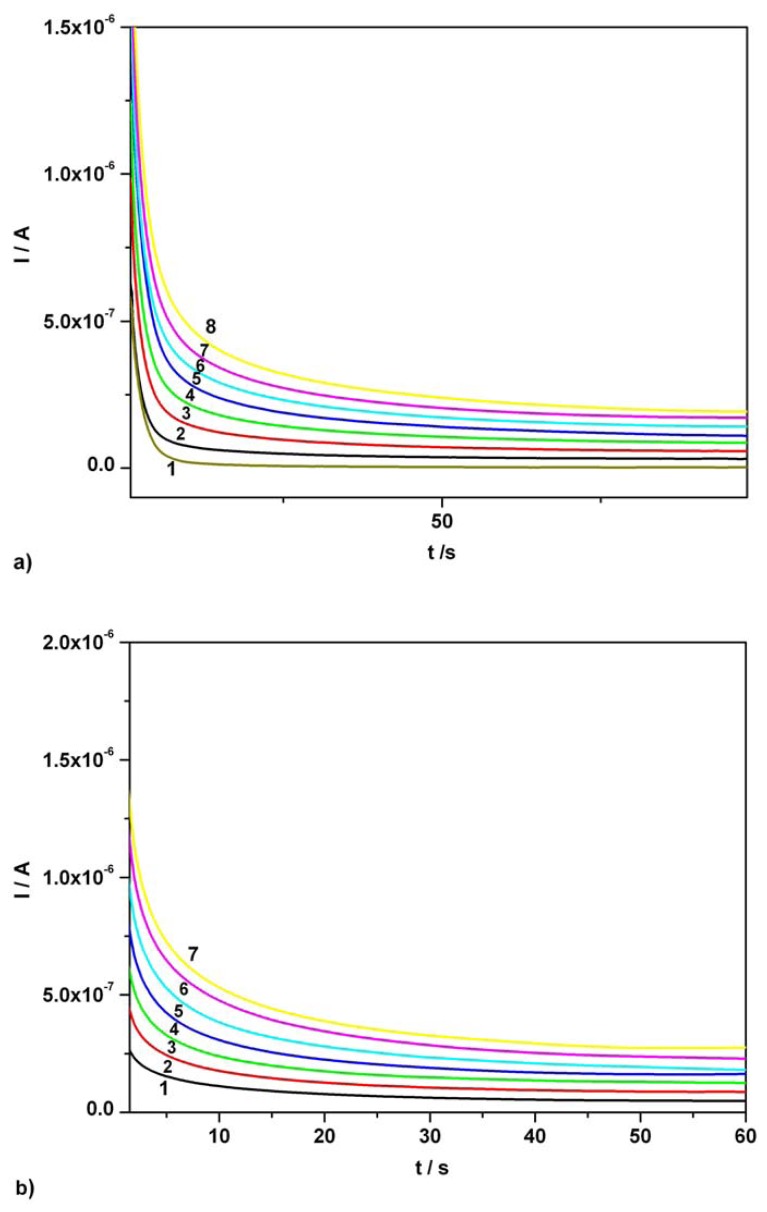
Chronoamperograms (CAs). **(a)** Effect of AA concentration at one potential level, + 0.5 V vs. SCE, around the corresponding current peak potential from CVs; 1 – supporting electrolyte; 2 – 0.01 mM AA; 3 – 0.02 mM AA; 4 – 0.03 mM AA; 5 – 0.04 mM AA; 6 – 0.05 mM AA; 7 – 0.06 mM AA; 8 – 0.07 mM AA; supporting electrolyte: Britton-Robinson buffer pH 1.96; **(b)** Effect of AC concentration at one potential level, + 0.75 V vs. SCE, around the corresponding current peak potential from CVs; 1 – 0.01 mM AC; 2 – 0.02 mM AC; 3 – 0.03 mM AC; 4 – 0.04 mM AC; 5 – 0.05 mM AC; 6 – 0.06 mM AC; 7 – 0.07 mM AC; supporting electrolyte: Britton-Robinson buffer pH 1.96.

Figure 5a presents a series of CAs obtained for AA over the concentration range 0.01 mM - 0.07 mM, in single component standard solution, at the potential level of + 0.5 V vs. SCE corresponding to AA anodic oxidation. The linear calibration data and LOD values are summarized in Table 1.

A similar evaluation, Figure 5b, corresponding to the concentration effect on the useful anodic response at BDDE was made for AC in standard solutions. The investigated compound was added in increasing concentration, from 0.01 mM to 0.07 mM and chronoamperograms were recorded at one potential level, + 0.75 V vs. SCE which corresponded to optimum AC anodic amperometric signal. Linear plot of anodic currents read at 50s (a sufficient time period for obtaining a steady state and a linearity) vs. AC concentration showed a very good correlation coefficient and a satisfactory high sensitivity (see also Table 1).

AA and AC simultaneous detection from di-component mixture of standard solutions followed.

**Figure 6.**
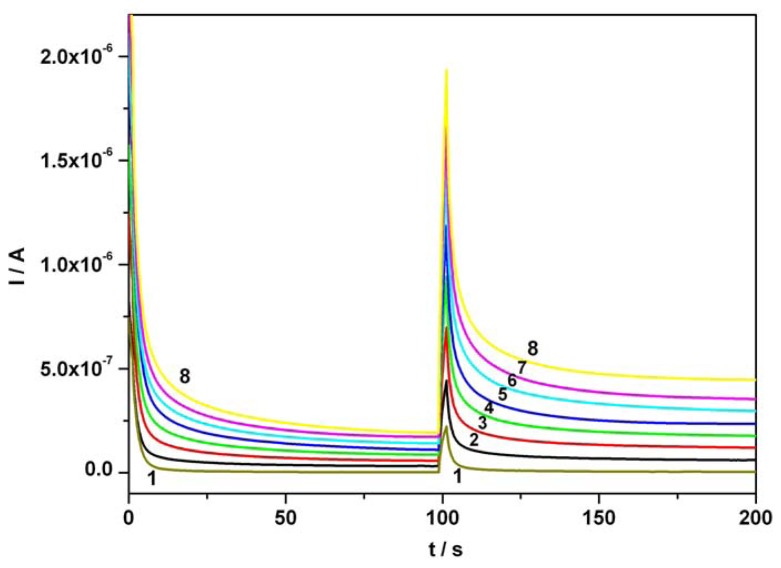
Continuous chronoamperograms (CAs) of AA and AC mixture at two potential levels, + 0.5 V and + 0.75 V vs. SCE, around the corresponding current peaks potentials from CVs; 1 – supporting electrolyte; 2 – 0.01 mM AA and 0.01 mM AC; 3 – 0.02 mM AA and 0.02 mM AC; 4 – 0.03 mM AA and 0.03 mM AC; 5 – 0.04 mM AA and 0.04 mM AC; 6 – 0.05 mM AA and 0.05 mM AC; 7 – 0.06 mM AA and 0.06 mM AC; 8 – 0.07 mM AA and 0.07 mM AC; supporting electrolyte: Britton-Robinson buffer pH 1.96.

Figure 6 illustrates a series of CAs obtained for the di-component system, at two potential levels, + 0.5 V and + 0.75 V vs. SCE, using for AA and AC equal concentrations ranged between 0.01 mM and 0.07 mM. Calibration plots of anodic currents at 100s for AA and 150s for AC versus concentration presented linearity, good correlation parameters and good sensitivities (see Table 1).

An extended remnant current signal of AA in the optimum potential range of AC can be observed when the mixture of investigated compounds is evaluated and therefore, an AA current correction becomes necessary. This correction required the subtraction of currents corresponding to AA oxidation (assumed steady state) and read at 100s, from the currents recorded for AC over the previously mentioned concentration range and read at 150s. Since the current due to AC presence in mixture at the optimum potential corresponding to AA sensing is practically the same as the background current of supporting electrolyte it does not influence the “resultant” amperometric signal corresponding to AA detection.

RSD ranged between 2 and 3% for situations involving both individual and simultaneous presence of AA and AC. LOD values have been calculated according to 3σ/slope criterion, where σ was estimated as the standard deviation applied to the net amperometric signal measured for the lowest analyte concentration corresponding to calibration plot. Under chronoamperometric conditions, LOD values obtained in single component systems have been practically recovered in di-component standard solutions.

Based on the general linearity and additivity of the chronoamperograms, a simple and fast amperometric way can also be used for the determination of a conventional global parameter as a preliminary general characteristic of the overall content in active substances of the system solution series resulted from the same mixture pharmaceutical product.

### 3.3. Chronoamperometric sequential and simultaneous determination of AA and AC from real samples

The exemplified simultaneous detection and the determination of AA and AC using the elaborated chronoamperometric method in association with standard addition and corresponding AA current corrections was achieved in real samples. Two UPSA pharmaceutical products, Efferalgan-effervescent tablets and Fervex – powder doses were used for preparation of the real samples as aqueous solutions.

**Figure 7.**
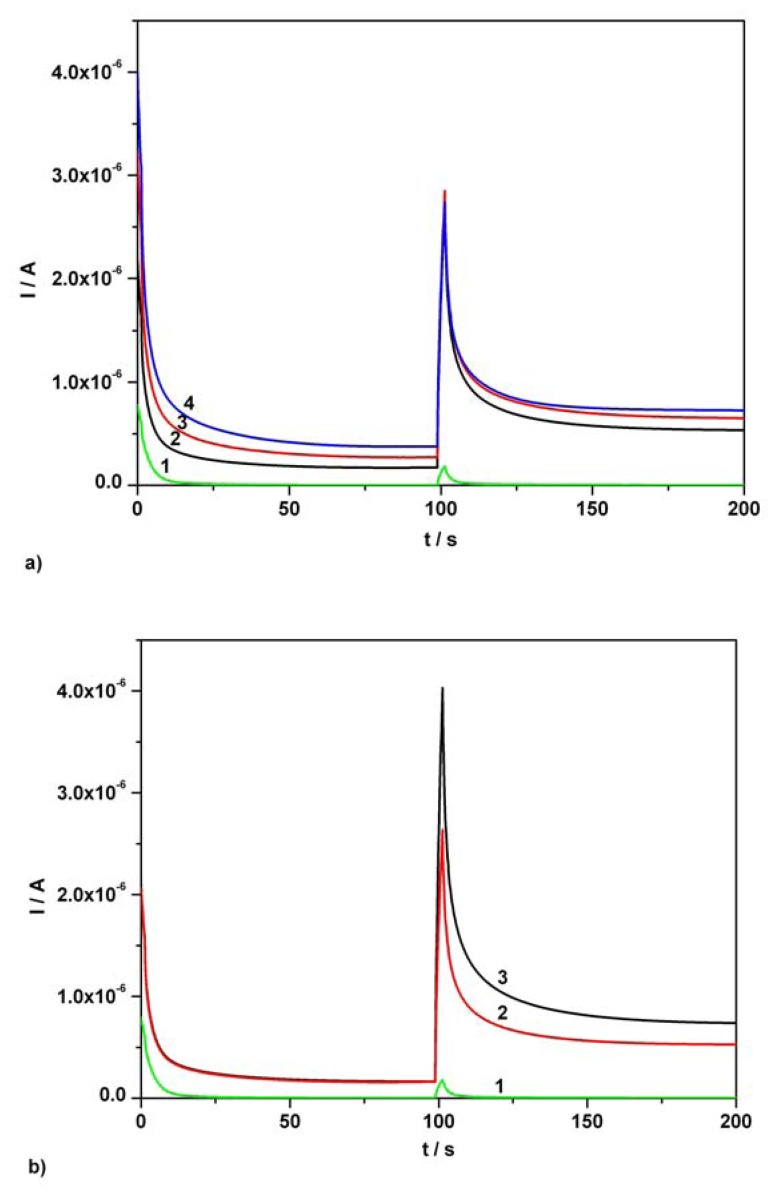
**(a)** Continuous chronoamperograms (CAs) at two potential levels, + 0.5 V, + 0.75 V vs. SCE. 1 – supporting electrolyte; 2 – 1/50 dilution of Efferalgan initial solution in supporting electrolyte; 3 - 1/50 dilution of Efferalgan initial solution in supporting electrolyte and AA addition, 0.03 mM AA final supplementary concentration; 4 - 1/50 dilution of Efferalgan initial solution in supporting electrolyte and AA addition, 0.06 mM AA final supplementary concentration; supporting electrolyte: Britton-Robinson buffer pH 1.96; current readings at 100s. **(b)** CAs at two potential levels, + 0.5 V, + 0.75 V vs. SCE. 1 - supporting electrolyte; 2 – 1/50 dilution of Efferalgan initial solution in supporting electrolyte; 3 - 1/50 dilution of Efferalgan initial solution in supporting electrolyte and AC addition, 0.06 mM AC final supplementary concentration; supporting electrolyte: Britton-Robinson buffer pH 1.96; current readings at 150s.

**Figure 8.**
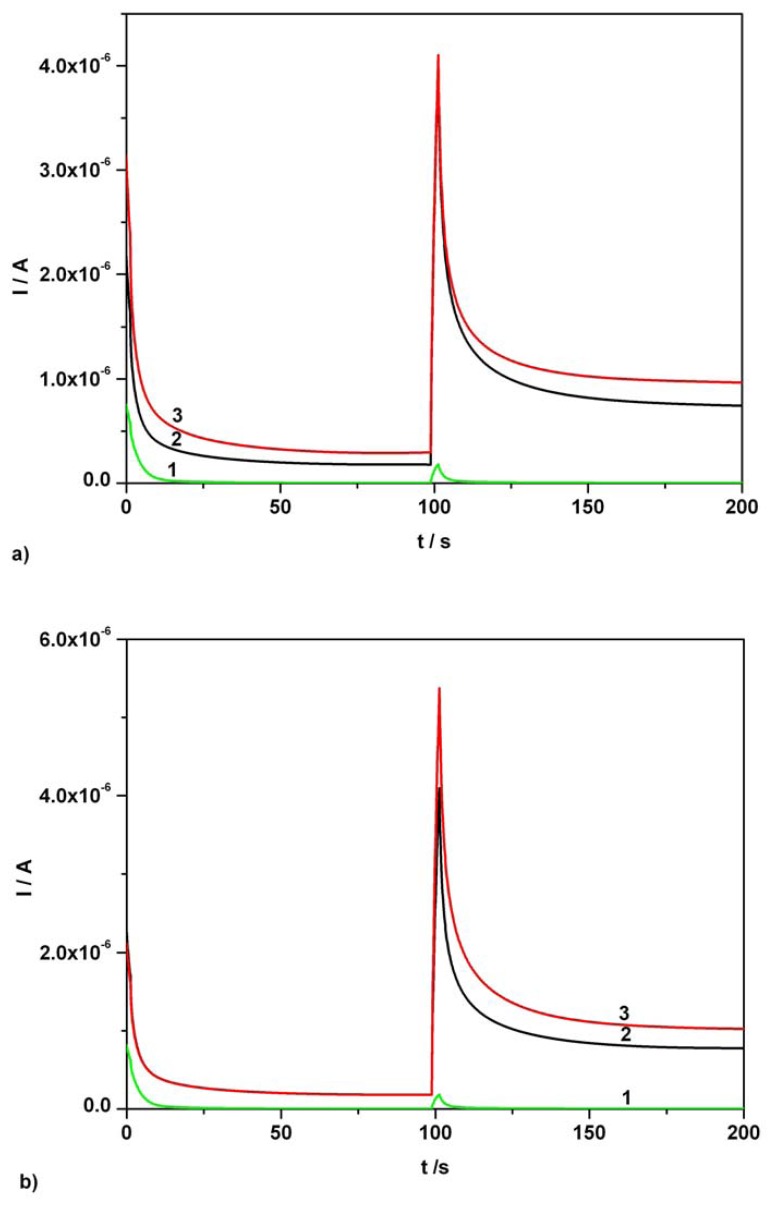
**(a)** Continuous chronoamperograms (CAs) at two potential levels, + 0.5 V, + 0.75 V vs. SCE. 1 – supporting electrolyte; 2 – 1/50 dilution of Fervex initial solution in supporting electrolyte; 3 - 1/50 dilution of Fervex initial solution in supporting electrolyte and AA addition, 0.03 mM AA final supplementary concentration; supporting electrolyte: Britton-Robinson buffer pH 1.96; current readings at 100s. **(b)** CAs at two potential levels, + 0.5 V, + 0.75 V vs. SCE. 1 – supporting electrolyte; 2 – 1/50 dilution of Fervex initial solution in supporting electrolyte; 3 - 1/50 dilution of Fervex initial solution in supporting electrolyte and AC addition, 0.06 mM AC final supplementary concentration; supporting electrolyte: Britton-Robinson buffer pH 1.96; current readings at 150s.

Illustrated in Figures 7a, 7b, 8a, and 8b, a number of CAs were recorded using real samples of aqueous solutions prepared under the conditions described in the experimental part and a pH 1.96 Britton-Robinson buffer as supporting electrolyte.

Figures 7a and 7b present a first example of simultaneous determination of AA and AC contents in an Efferalgan real sample solution. In order to prepare the aqueous real sample solution, the volume condition mentioned in the experimental part was followed and a 2.8787 g Efferalgan tablet was used. The very small volumes corresponding to final concentrations of 0.03 mM and 0.06 mM AA standard solutions were added in a real sample resulted from 1 mL Efferalgan initial solution diluted to 50 mL final volume with Britton-Robinson supporting electrolyte (1/50 dilution). The resulted chronoamperograms are depicted in Figure 7a. Determination of AC was accomplished when a very small volume corresponding to final concentration of 0.06 mM AC standard solution was added to the same overall volume of real sample (Figure 7b). Using standard addition method, the determined average contents were 203.9 mg AA / tablet and 346.9 mg AC / tablet.

It should be noted that the 206.3 mg AA and 344.8 mg AC values per tablet represent the average contents determined when five Efferalgan effervescent tablets with an average weight of 2.8813 g were investigated. AA and AC contents values in Efferalgan tablets indicated by the UPSA supplier were 200 mg AA / tablet and 330 mg AC / tablet respectively. In order to determine a correct average content of AC in the pharmaceutical tablets used, the same type of AA current correction as in the case of investigated mixture of standard solutions was applied.

The second example concerning AA and AC simultaneous determination in pharmaceutical formulations is connected with the use of a Fervex aqueous solution obtained from a 13.3882 g Fervex powder dose using the volume control regime described in the experimental section. A 1 mL Fervex initial solution diluted to 50 mL volume with supporting electrolyte constituted the final volume of the real sample.

Figure 8a shows the chronoamperograms recorded at two potential levels for individual real sample and for the mixture containing supplementary 0.03 mM added AA standard solution (curve 3) in investigated dilute real sample and dilute real sample (curve 2). A new very small volume addition corresponding to 0.06 mM final concentration of AC in 50mL final volume dilute Fervex real sample gave rise to continuous chronoamperograms, curve 3 compared to curve 2, illustrated in Figure 8b. The determined average content corresponding to the chosen example was of 203.9 mg AA / dose and 519.1 mg AC per dose, respectively. Investigating five Fervex doses having the average weight of 13.3178 g, the application of standard addition method gave rise to determined average contents of 200.2 mg AA per dose and 510.7 mg AC per dose. The average content of AC in the above mentioned doses was determined using the same kind of AA current correction as in the case of Efferalgan sample. According to the UPSA product specification one Fervex powder dose contains 200 mg ascorbic acid and 500 mg acetaminophen. Thus, the average analytical data obtained for both investigated pharmaceutical preparations corresponded to very good degrees of component recovery without evidence of major matrix effects.

The obtained data confirmed our previous conclusions concerning the adequacy, viability and flexibility in the use of commercial BDDE for the individual sensing and simultaneous determination of various analytes in different circumstances [49, 53].

Figure 8a shows the chronoamperograms recorded at two potential levels for individual real sample and for the mixture containing supplementary 0.03 mM added AA standard solution (curve 3) in investigated dilute real sample and dilute real sample (curve 2). A new very small volume addition corresponding to 0.06 mM final concentration of AC in 50mL final volume dilute Fervex real sample gave rise to continuous chronoamperograms, curve 3 compared to curve 2, illustrated in Figure 8b. The determined average content corresponding to the chosen example was of 203.9 mg AA / dose and 519.1 mg AC per dose, respectively. Investigating five Fervex doses having the average weight of 13.3178 g, the application of standard addition method gave rise to determined average contents of 200.2 mg AA per dose and 510.7 mg AC per dose. The average content of AC in the above mentioned doses was determined using the same kind of AA current correction as in the case of Efferalgan sample. According to the UPSA product specification one Fervex powder dose contains 200 mg ascorbic acid and 500 mg acetaminophen. Thus, the average analytical data obtained for both investigated pharmaceutical preparations corresponded to very good degrees of component recovery without evidence of major matrix effects.

The obtained data confirmed our previous conclusions concerning the adequacy, viability and flexibility in the use of commercial BDDE for the individual sensing and simultaneous determination of various analytes in different circumstances [49, 53].

## 4. Conclusions

The anodic CV and CA data were obtained for individual and mixture standard solutions of ascorbic acid and acetaminophen at unmodified BDDE in acidic buffered media. The optimum potential values previously defined from voltammetric data for anodic amperometric sensing were applied as fixed potential in the systematic application of a long time chronoamperometry method for calibration and practical uses.

Very good linearities of the calibration plots of anodic current versus concentration of the investigated electrochemical substances resulted from CV data of di-component, from CA data of single component, and continuous CA data of di-component standard solutions systems. Adjacent analytical data regarding RSD, LOD and sensitivities were obtained.

A new effective anodic chronoamperometric method for the sequential two-step potential electrochemical sensing and simultaneous determination of ascorbic acid and acetaminophen using an unmodified commercial BDDE in buffered aqueous solutions has been elaborated.

The chronoamperometric method coupled with the standard addition has been successfully used for a fast analytical evaluation of pharmaceutical formulations which contain both ascorbic acid and acetaminophen without major matrix effects. The average content values of AA and AC in UPSA effervescent tablets and powder doses, explored as real samples, were measured in good accordance with those indicated by the supplier, and our previous data [53].

Based on the general linearity of the current-concentration calibration plots, the direct amperometric method using stabilized commercial BDDE as a sensor could also be employed for individual sensing in single component systems. At the same time, based on the additive features of the amperometric signals, a simple and fast amperometric detection could also be estimated as a first “pattern finger” characterization of the overall content in active substances, AA and AC, in the series of the same product type regarded as mixture system series.

## Figures and Tables

**Table 1.** Parameters of the calibration plots (I = aC + b; a (μA / mM); C (mM); b (μA)), correlation coefficients and LOD values for ascorbic acid and acetaminophen determination using chronoamperometry; [*] – single component solution; [**] – di-component solution.

***Component***	***Figure***	***Concentration range (mM)***	***Regression equation of linear calibration plot***	***Sensitivity (μA/mM)***	***R****^2^*	***LOD (μM)***
Ascorbic acid	Fig.5a [*]^1^	0.01-0.07	I = 3.332C + 0.0002	3.332	0.999	0.80
Ascorbic acid	Fig.5a [*]^2^	0.01-0.07	I = 3.343C + 0.009	3.343	0.995	0.86
Acetaminophen	Fig. 5b [*]	0.01-0.07	I = 3.699C + 0.001	3.699	0.997	0.97
Ascorbic acid	Fig. 6 [**]	0.01-0.07	I = 3.343C + 0.009	3.343	0.995	0.86
Acetaminophen	Fig. 6 [**]	0.01-0.07	I = 3.806C - 0.006	3.806	0.994	1.42

1 – current readings at 50s;

2 – current readings at 100s.
